# Effect of Bariatric Surgery on Osteoarthritis‐Related Pain and Function: A Systematic Review and Meta‐Analysis

**DOI:** 10.1002/wjs.70236

**Published:** 2026-01-21

**Authors:** Mohammed Mana Mohammed Alqahtani, António Raposo, Abdullah Mohammed Saeed Alshalaan, Saeed Mohammed Saad Asiri, Jaber Salman Yazeed Alfaifi, Saleh Mohammed Saleh Alqahtani, Rawan Asad Salameh Shawabkeh, Afnan Ali Saeed Abonukhaa, Sara Ahmed Elmahadi Ahmed, Maha Hassan Ali Asiri, Mohammad Abdullah Saeed Almastoor, Ariana Saraiva, Ashwag Saleh Alsharidah, Ibrahim Yahya Al Yasin, Najim Z. Alshahrani

**Affiliations:** ^1^ Department of Endocrine Medicine Armed Forces Hospital Southern Region (AFHSR) Khamis Mushit Aseer Region Saudi Arabia; ^2^ CBIOS (Research Center for Biosciences and Health Technologies) ECTS (School of Health Sciences and Technologies) Lusófona University Lisbon Portugal; ^3^ Najran Armed Forces Hospital Najran Saudi Arabia; ^4^ Department of Internal Medicine Armed Forces Hospital Southern Region (AFHSR) Khamis Mushit Aseer Region Saudi Arabia; ^5^ Abha Rehabilitation Center Ministry of Human Resources and Social Development (MHRSD) Abha Saudi Arabia; ^6^ Faculty of Veterinary Medicine Research in Veterinary Medicine (I‐MVET) Lisbon University Centre Lusófona University Lisbon Portugal; ^7^ Faculty of Veterinary Medicine Veterinary and Animal Research Centre (CECAV) Lisbon University Centre Lusófona University Lisbon Portugal; ^8^ Department of Physiology College of Medicine Qassim University Buraydah Saudi Arabia; ^9^ Clinical Nutrition Department College of Nursing and Health Sciences Jazan University Jazan Saudi Arabia; ^10^ Department of Family and Community Medicine, Faculty of Medicine University of Jeddah Jeddah Saudi Arabia

**Keywords:** bariatric surgery, meta‐analysis, obesity, osteoarthritis, pain and function

## Abstract

**Aim:**

This systematic review and meta‐analysis aimed to synthesize current evidence on the effect of metabolic and bariatric surgery on osteoarthritis (OA) diagnosis, pain, stiffness, and functional outcomes.

**Method:**

A systematic search was conducted in PubMed/MEDLINE, Scopus, Web of Science, and the Cochrane Library for studies published between January 2000 and July 2025. Eligible studies included adult patients undergoing any form of bariatric surgery who had either a documented diagnosis of OA before and after surgery or a quantitative assessment of OA‐related symptoms using validated instruments such as the Western Ontario and McMaster Universities Osteoarthritis Index (WOMAC). Data were pooled using random‐effects models, and heterogeneity was assessed using the I^2^ statistic.

**Results:**

Twelve studies published between 2007 and 2024 met the inclusion criteria, encompassing 12,000 participants across prospective and retrospective cohorts. The pooled odds ratio for OA diagnosis after surgery compared with preoperative status was 0.21 (95% CI: 0.11–0.41), indicating a 79% reduction in OA likelihood. Significant improvements were observed in WOMAC pain, stiffness, and physical function scores at 6 and 12 months postoperatively, with overall pooled mean differences of −20.80 (95% CI: −32.74 to −8.86) and −17.12 (95% CI: −25.28 to −8.96), respectively. Heterogeneity was substantial across studies (I^2^ > 75%).

**Conclusions:**

Metabolic and bariatric surgery is associated with significant reductions in osteoarthritis diagnosis and improvements in OA‐related pain and physical function. These findings suggest that surgical weight loss may provide meaningful benefits for joint health in patients with obesity.

## 1 Introduction

According to the Global Burden of Disease Study 2021, osteoarthritis (OA) is a prevalent musculoskeletal condition that affects nearly 528 million people worldwide, corresponding to approximately 23% of the global population aged ≥ 40 years [1]. It represents a leading cause of disability and pain, primarily involving weight‐bearing joints such as the knees and hips [1]. The burden of OA has risen markedly over recent decades, driven by increasing life expectancy, sedentary lifestyles, and the global obesity epidemic [2]. Excess body weight is one of the most significant modifiable risk factors for the onset and progression of OA, contributing both to mechanical overloading of joints and to systemic metabolic and inflammatory dysregulation [3].

Obesity and OA share overlapping pathophysiological mechanisms involving mechanical stress, low‐grade systemic inflammation, oxidative stress, and altered adipokine signaling [3, 4]. Adipose tissue functions as an active endocrine organ that secretes proinflammatory cytokines, including interleukin‐6 and tumor necrosis factor‐α, which can accelerate cartilage degradation and synovial inflammation [4, 5]. These processes not only exacerbate pain and functional impairment but also contribute to disease progression and increased health care burden.

Metabolic and bariatric surgery has emerged as the most effective intervention for achieving substantial and sustained weight loss in individuals with obesity [6]. Procedures such as Roux‐en‐Y gastric bypass (RYGB), laparoscopic sleeve gastrectomy (LSG), and laparoscopic adjustable gastric banding (LAGB) promote weight reduction through restrictive, malabsorptive, and neurohormonal mechanisms [7]. Beyond their metabolic effects, these procedures induce favorable changes in systemic inflammation, insulin sensitivity, and lipid metabolism, which may confer additional benefits for joint health and musculoskeletal function [8].

Several clinical studies have demonstrated significant improvements in OA‐related outcomes after bariatric surgery [9, 10]. Reductions in body weight have been associated with decreased joint pain, improved physical function, and enhanced quality of life, as measured by validated tools such as the Western Ontario and McMaster Universities Osteoarthritis Index (WOMAC) Index [10]. Longitudinal studies have further indicated that these benefits may persist over time, suggesting both symptomatic and potential structural improvements, including increases in joint space width [9, 11].

Despite these promising findings, evidence across individual studies remains heterogeneous, with variations in study design, surgical procedure, follow‐up duration, and outcome assessment. Prior systematic reviews have suggested that bariatric surgery may reduce OA symptoms and delay disease progression, yet a comprehensive quantitative synthesis of these effects remains limited. Therefore, this systematic review and meta‐analysis was conducted to evaluate the impact of metabolic and bariatric surgery on osteoarthritis‐related pain and functional outcomes, providing an updated and rigorous assessment of available evidence.

## 2 Methods

### 2.1 Study Design

This systematic review and meta‐analysis was conducted to synthesize available evidence on the impact of bariatric surgery on osteoarthritis outcomes. The study protocol was designed in accordance with the Cochrane Handbook for Systematic Reviews of Interventions and is reported following the Preferred Reporting Items for Systematic reviews and Meta‐Analyses (PRISMA) 2020 guidelines. The study was structured using the PICO framework:

- Population (P): Adult patients with obesity.
- Intervention (I): Bariatric surgery, including techniques such as laparoscopic sleeve gastrectomy (LSG), Roux‐en‐Y gastric bypass (RYGB), and laparoscopic adjustable gastric banding (LAGB).
- Comparison (C): Preoperative versus postoperative status.
- Outcome (O): Changes in osteoarthritis‐related outcomes, including (1) prevalence or odds of a clinical diagnosis of osteoarthritis before and after surgery, and (2) changes in osteoarthritis‐related pain, stiffness, and physical function as measured by validated tools such as WOMAC.

### 2.2 Search Strategy

A comprehensive literature search was performed across four electronic databases: PubMed/MEDLINE, Scopus, Web of Science (WOS), and the Cochrane Library. The search covered all studies published from January 2000 to July 2025. The search strategy combined Medical Subject Headings (MeSH) and free‐text keywords related to bariatric surgery (e.g., “Bariatric Surgery,” “Gastric Bypass,” “Sleeve Gastrectomy”) and osteoarthritis (e.g., “Osteoarthritis,” “Joint Pain,” “Degenerative Joint Disease”). Boolean operators (AND/OR) were used to combine the search terms. The search was restricted to articles published in the English language. A full detailed list of the search strategies for each database is available in Supporting Information S1.

### 2.3 Eligibility Criteria

Studies were eligible for inclusion if they were observational in design, either prospective or retrospective cohort studies, or clinical trials involving adult patients who underwent any form of bariatric surgery. To be included, studies were required to report at least one of the primary outcomes of interest. These outcomes included either the number or proportion of patients with a confirmed diagnosis of osteoarthritis before and after surgery or quantitative assessments of osteoarthritis‐related symptoms such as pain, stiffness, and physical function. Symptom outcomes were considered eligible when measured using validated instruments, including the WOMAC Index or comparable scales, and when accompanied by appropriate measures of variance at baseline and at a minimum follow‐up period of 6 months postoperatively. Studies were excluded if they were review articles, case reports, editorials, commentaries, or conference abstracts. In addition, studies that did not provide sufficient quantitative data to calculate an effect size, such as an odds ratio or mean difference, were excluded from the analysis.

### 2.4 Study Selection and Screening

All records identified through the database searches were imported into Rayyan (https://www.rayyan.ai/) for duplicate removal and management. Four reviewers independently screened the titles and abstracts of all unique records against the predefined eligibility criteria. Subsequently, the full texts of potentially relevant articles were retrieved and assessed for final inclusion by the same four reviewers. Any disagreements during the screening process were resolved through discussion and consensus; if a consensus could not be reached, a senior reviewer was consulted for a final decision.

### 2.5 Data Extraction

Data were independently extracted from the included studies by four reviewers using a standardized data extraction form created in Microsoft Excel. The extracted information included study characteristics (first author, publication year, and study design), participant demographics (total sample size, number of patients with osteoarthritis, mean age, mean BMI, percentage of females), intervention details (type and number of surgical procedures performed), and primary outcome data. For the binary outcome, the number of patients with an osteoarthritis diagnosis before and after surgery was extracted. For continuous outcomes, the mean WOMAC scores for pain, stiffness, and physical function, along with their standard deviations and sample sizes, were extracted at 6‐month and 12‐month follow‐up periods.

### 2.6 Quality Assessment

The methodological quality and risk of bias of the included nonrandomized studies were evaluated using the Risk Of Bias In Non‐randomized Studies – of Interventions (ROBINS‐I) tool, developed by the Cochrane Bias Methods Group [12]. This tool assesses potential sources of bias across seven distinct domains: (1) bias due to confounding, (2) bias in the selection of participants into the study, (3) bias in the classification of interventions, (4) bias due to deviations from intended interventions, (5) bias due to missing data, (6) bias in the measurement of outcomes, and (7) bias in the selection of the reported result.

Each study was carefully reviewed and assigned a judgment for each domain as low, moderate, serious, or critical risk of bias, according to the ROBINS‐I guidance. The overall risk of bias for each study was then determined based on the highest level of risk observed in any of the individual domains. The assessment was performed independently by two reviewers to minimize subjectivity and enhance reliability. In cases where discrepancies arose between reviewers, these were resolved through discussion and consensus, with the involvement of a third reviewer when necessary.

### 2.7 Statistical Analysis

All statistical analyses were performed using R (version 4.3.3) with the metafor package. A random‐effects model was used for all meta‐analyses to account for anticipated clinical and methodological heterogeneity between studies. For the dichotomous outcome of osteoarthritis diagnosis, pooled odds ratios (ORs) with 95% confidence intervals (CIs) were calculated. For the continuous outcome of WOMAC scores, pooled mean differences (MD) with 95% CIs were calculated for each subscale (pain, stiffness, and physical function) at different follow‐up intervals. Statistical heterogeneity was assessed using Cochran's Q test and quantified with the I^2^ statistic. An I^2^ value greater than 75% was considered indicative of substantial heterogeneity. The results of the meta‐analyses were visualized using forest plots. A *p*‐value of less than 0.05 was considered statistically significant for all analyses.

## 3 Results

### 3.1 Study Selection

**FIGURE 1:**
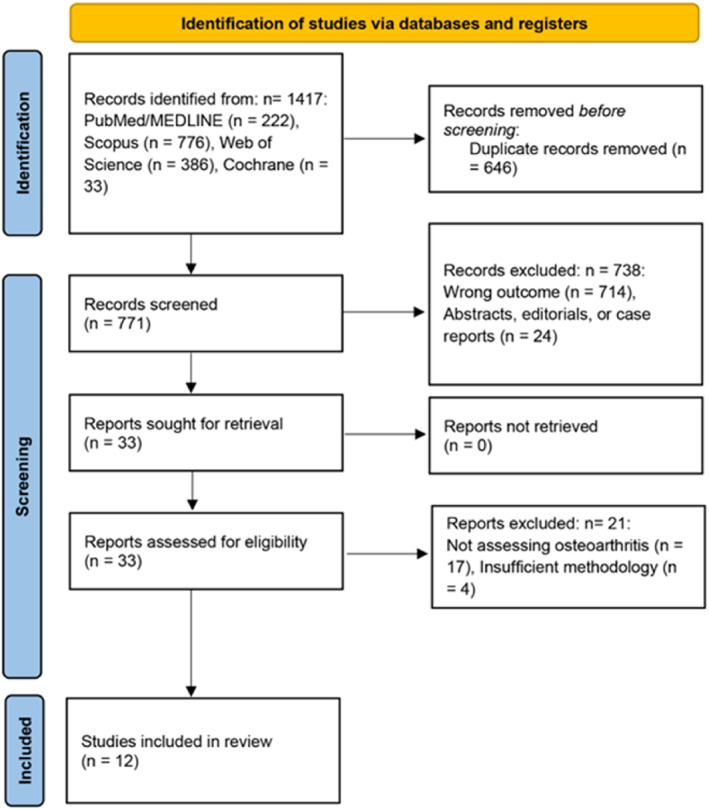
PRISMA 2020 flow diagram of study selection.

The initial database search identified a total of 1417 records. After removing 646 duplicates, 771 unique records were screened by title and abstract. During this screening process, 738 articles were excluded because they addressed the wrong outcome, were abstracts or editorials, or were case reports. The full texts of the remaining 33 reports were assessed for eligibility. Of these, 21 reports were excluded for either not assessing osteoarthritis (*n* = 17) or having insufficient methodology (*n* = 4). Ultimately, 12 studies met the full eligibility criteria and were included in the final systematic review (Figure 1).

### 3.2 Baseline Characteristics of the Included Studies

A total of 12 studies, published between 2007 and 2024, were included in this systematic review and meta‐analysis (Table 1). The studies comprised both retrospective (*n* = 6) and prospective (*n* = 6) cohort designs, with maximum follow‐up durations ranging from 12 to 192 months. The mean age of participants varied from 34.0 to 46.9 years, and the mean preoperative BMI ranged from 33.1 to 53.1 kg/m^2^, reflecting populations with obesity or severe obesity. Across all studies, the majority of participants were female, with proportions ranging from 67.0% to 84.3%.

**TABLE 1:** Baseline characteristics of the included studies.

Study	Design	Patients with osteoarthritis, *n* (% before surgery)	Follow‐up (months)§	Age (years)†	BMI (kg/m^2^)†	Female, *n* (%)	LSG, *n* (%)	RYGB, *n* (%)	LAGB, *n* (%)	Other techniques, *n* (%)
Paran et al. (2007) [13]	Retrospective cohort	23 (20.0%)	192	39.0	47.0	87 (75.7)	NR	NR	NR	115 (100)
Peluso et al. (2007) [14]	Retrospective cohort	216 (54.0%)	30.6	44.8	48.0	337 (84.3)	NR	400 (100)	NR	NR
Sultan et al. (2009) [15]	Prospective cohort	11 (20.8%)	24	46.9	33.1	44 (83.0)	NR	NR	53 (100)	NR
Gianos et al. (2012) [16]	Retrospective cohort	8 (19.0%)	72	NA	33.9	30 (71.4)	24 (57.1)	8 (19.0)	10 (23.8)	NR
Yaghoubian et al. (2012) [17]	Retrospective cohort	116 (21.6%)	12	46.7‡	45.6‡	NA	192 (35.8)	345 (64.3)	NR	NR
Mohos et al. (2014) [18]	Prospective cohort	64 (72.7%)	39	45.8	42.5	64 (72.7)	NR	NR	88 (100)	NR
Hunter et al. (2016) [19]	Prospective cohort	672 (61.8%)	120	41.4	53.1	NA	NR	1087 (100)	NR	NR
Juodeikis et al. (2019) [20]	Prospective cohort	71 (68.9%)	60	45.9	47.5	69 (67.0)	NR	NR	103 (100)	NR
Neuberg et al. (2020) [21]	Retrospective cohort	31 (19.0%)	96	41.0	40.3	129 (79.6)	NR	NR	NR	163 (100)
Level et al. (2021) [22]	Prospective cohort	38 (19.0%)	60	36.9	41.8	110 (82.7)	63 (47.4)	70 (52.6)	NR	NR
Rheinwalt et al. (2022) [23]	Prospective cohort	72 (58.5%)	24	44.0	42.0	90 (73.2)	NR	68 (55.3)	NR	55 (44.7)
Kehagias et al. (2024) [24]	Retrospective cohort	7 (6.7%)	188	34.0	43.4	82 (78.9)	104 (100)	NR	NR	NR

*Note:* †mean. ‡median. §maximum follow‐up.

Abbreviations: BMI, body mass index; LAGB, laparoscopic adjustable gastric banding; LSG, laparoscopic sleeve gastrectomy; NA, not available; NR, not reported; RYGB, Roux‐en‐Y gastric bypass.

The prevalence of osteoarthritis prior to surgery varied widely, from 6.7% (Kehagias et al., 2024) to 72.7% (Mohos et al., 2014). Various bariatric techniques were represented, including laparoscopic sleeve gastrectomy (LSG), Roux‐en‐Y gastric bypass (RYGB), laparoscopic adjustable gastric banding (LAGB), and other less common procedures. RYGB and LAGB were the most frequently reported surgical types, accounting for the entire surgical sample in several studies (Peluso et al., 2007; Hunter et al., 2016; Mohos et al., 2014; Juodeikis et al., 2019). Only a few studies included mixed surgical cohorts (e.g., Gianos et al., 2012; Level et al., 2021; Rheinwalt et al., 2022), whereas others focused on a single procedure type.

### 3.3 Impact of Bariatric Surgery on Osteoarthritis Diagnosis

**FIGURE 2:**
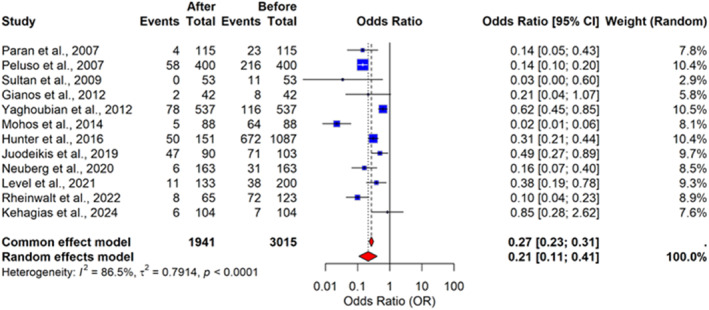
Forest plot of the odds ratios for osteoarthritis diagnosis after versus before bariatric surgery.

The meta‐analysis evaluated the odds of osteoarthritis diagnosis after bariatric surgery compared with the preoperative period (Figure 2). The pooled analysis using a random‐effects model showed a statistically significant reduction in the likelihood of osteoarthritis after surgery. The pooled odds ratio (OR) was 0.21 (95% confidence interval [CI]: 0.11–0.41), indicating that patients had approximately 79% lower odds of receiving an osteoarthritis diagnosis after bariatric surgery. Considerable heterogeneity was observed across studies (I^2^ = 86.5%, *p* < 0.0001), suggesting variability in study design, populations, and follow‐up duration.

### 3.4 Assessment of Publication Bias

**FIGURE 3:**
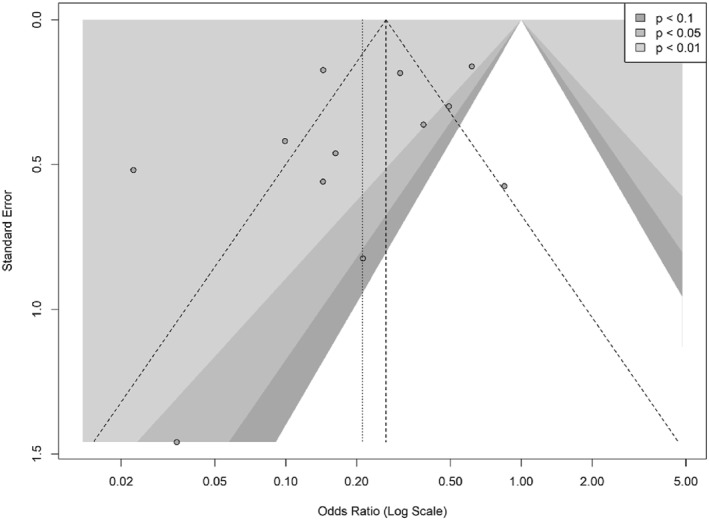
Funnel plot with contour enhancement for the evaluation of publication bias in studies of bariatric surgery and osteoarthritis.

Publication bias was assessed using a contour‐enhanced funnel plot and Egger's regression test (Figure 3). Visual inspection of the funnel plot indicated slight asymmetry, suggesting a potential underrepresentation of smaller studies with nonsignificant results. However, Egger's regression test did not confirm the presence of publication bias (*t* = −1.19, *p* = 0.26). Therefore, although minor visual asymmetry was observed, there was no statistically significant evidence of publication bias in this meta‐analysis.

### 3.5 WOMAC Score Improvement at 6 Months

**FIGURE 4:**
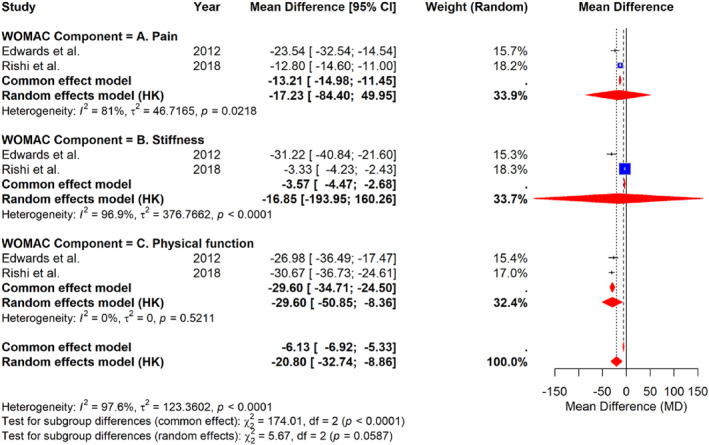
Forest plot of the mean difference in WOMAC scores at 6 months after bariatric surgery, grouped by subscale (pain, stiffness, and physical function).

At 6 months after surgery, pooled analyses showed significant improvements in WOMAC scores for pain, stiffness, and physical function (Figure 4). For the pain subscale, the pooled mean difference (MD) was −17.23 (95% CI: −84.40 to 49.95) with substantial heterogeneity (I^2^ = 81%, *p* = 0.0218). The stiffness subscale showed a pooled MD of −16.85 (95% CI: −193.95 to 160.26) and very high heterogeneity (I^2^ = 96.9%, *p* < 0.0001). The most consistent improvement was observed in physical function, with a pooled MD of −29.60 (95% CI: −50.85 to −8.36) and no evidence of heterogeneity (I^2^ = 0%, *p* = 0.5211). The overall pooled mean difference across all WOMAC components was −20.80 (95% CI: −32.74 to −8.86), confirming a significant improvement in osteoarthritis symptoms at 6 months postoperatively.

### 3.6 WOMAC Score Improvement at 12 Months

**FIGURE 5:**
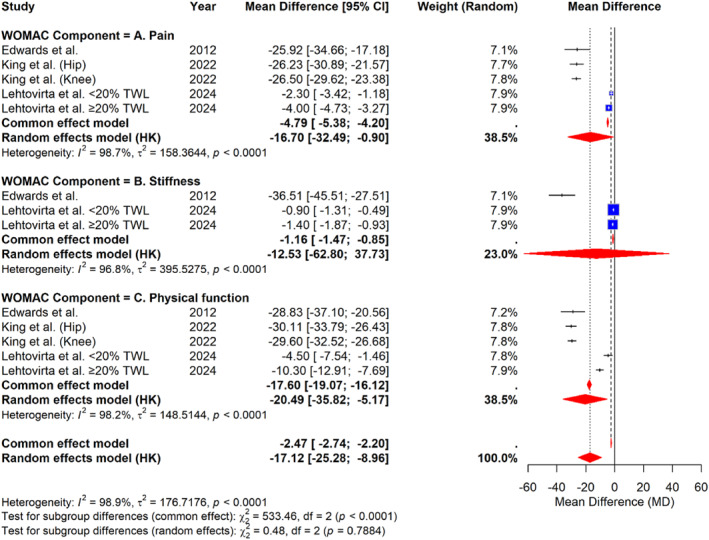
Forest plot of the mean difference in WOMAC scores at 12 months after bariatric surgery, grouped by subscale (pain, stiffness, and physical function).

At the 12‐month follow‐up, patients continued to show significant and sustained improvements in osteoarthritis symptoms across all WOMAC subscales (Figure 5). For the pain subscale, the pooled analysis showed a mean difference (MD) of −16.70 (95% CI: −32.49 to −0.90), with very high heterogeneity (I^2^ = 98.7%, *p* < 0.0001). The stiffness subscale demonstrated a mean difference of −12.53 (95% CI: −62.80 to 37.73), also with substantial heterogeneity (I^2^ = 96.8%, *p* < 0.0001). The most pronounced improvement was observed in the physical function subscale, with a pooled MD of −20.49 (95% CI: −35.82 to −5.17) and very high heterogeneity (I^2^ = 98.2%, *p* < 0.0001). The overall pooled mean difference across all WOMAC components was −17.12 (95% CI: −25.28 to −8.96), indicating a significant and sustained improvement in osteoarthritis‐related symptoms 1 year after bariatric surgery, despite considerable heterogeneity across the included studies (overall I^2^ = 98.9%, *p* < 0.0001).

### 3.7 Risk of Bias Assessment

**FIGURE 6:**
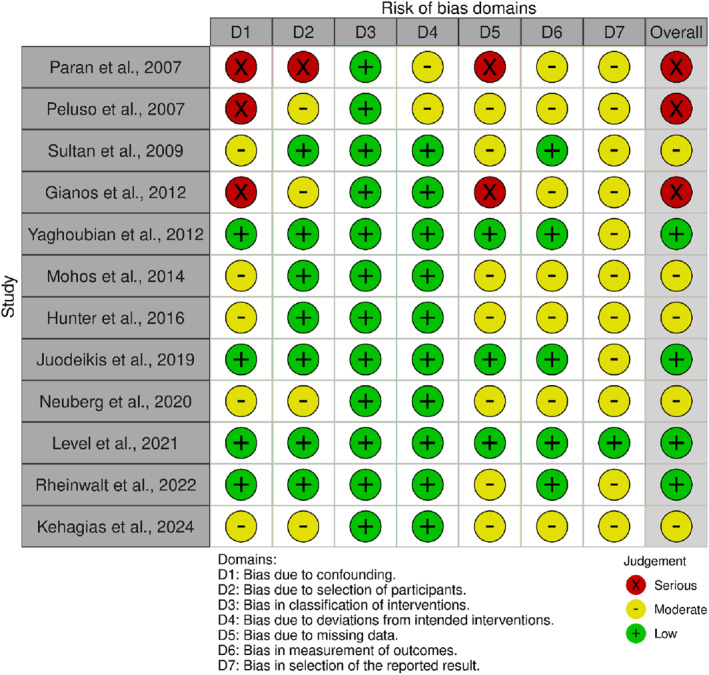
Risk of bias assessment using the ROBINS‐I tool.

The ROBINS‐I assessment (Figure 6) demonstrated that the overall risk of bias across the included studies ranged from low to serious. Three studies (Paran et al., 2007; Peluso et al., 2007; Gianos et al., 2012) were judged to have a serious overall risk of bias. Five studies were classified as having a moderate risk, and the remaining four were rated as having a low risk of bias. The most frequent sources of a 'serious' rating were bias due to confounding (D1) and missing data (D5), reflecting limitations in study design and follow‐up. Conversely, bias in the classification of interventions (D3) was consistently low across all studies. Bias in the measurement of outcomes (D6) was generally rated as low or moderate, and bias from the selection of reported results (D7) was typically moderate.

## 4 Discussion

This systematic review and meta‐analysis synthesized evidence from 12 studies evaluating the impact of bariatric surgery on osteoarthritis‐related outcomes. The findings demonstrate a significant reduction in osteoarthritis diagnosis rates and consistent improvements in pain, stiffness, and physical function after bariatric surgery. The pooled analysis revealed a 79% decrease in the odds of osteoarthritis diagnosis after surgery, alongside clinically meaningful improvements in the WOMAC Index subscales at both 6 and 12 months. These results collectively suggest that bariatric surgery may have a favorable effect on osteoarthritis symptoms and functional status among patients with obesity.

The observed reductions in osteoarthritis prevalence after bariatric surgery are consistent with previous reviews that reported substantial improvements in musculoskeletal and rheumatic disease outcomes after weight loss. De Souza et al. (2025) demonstrated that metabolic surgery was associated with improvements in several obesity‐related rheumatic conditions, including OA, gout, and fibromyalgia, highlighting the broader systemic benefits of surgical weight reduction [10]. Similarly, Gallo et al. (2018) described reductions in inflammatory biomarkers and improved rheumatic disease activity scores after bariatric surgery, supporting the notion that surgery may exert disease‐modifying effects beyond mechanical load reduction [25].

The improvements observed in WOMAC pain and function scores in the present analysis are aligned with prior prospective studies. King et al. (2022) reported that more than 60% of patients undergoing Roux‐en‐Y gastric bypass or sleeve gastrectomy experienced sustained improvements in bodily pain and physical function up to 7 years postoperatively [9]. Similarly, Edwards et al. (2012) and Gill et al. (2011) documented significant reductions in knee pain and stiffness within 1 year after surgery, accompanied by improvements in quality of life and radiographic joint parameters [11, 26]. The persistence of these benefits across different surgical procedures and time frames underscores the robustness of the association between weight loss and symptom improvement in OA.

The mechanisms underlying these clinical benefits are likely multifactorial. The most direct mechanism is mechanical unloading, as reductions in body weight led to proportional decreases in joint compressive forces [7]. It is estimated that each kilogram of body weight lost reduces the load on the knee joint by approximately fourfold during ambulation [27]. This biomechanical effect contributes to pain reduction and improved joint mobility, particularly in weight‐bearing joints. Beyond mechanical factors, metabolic and inflammatory changes after bariatric surgery likely play an equally important role [28]. Weight loss is associated with decreases in circulating proinflammatory cytokines, including interleukin‐6 (IL‐6) and tumor necrosis factor‐α (TNF‐α), both of which contribute to cartilage degradation and synovial inflammation [29]. Furthermore, bariatric surgery enhances insulin sensitivity and alters adipokine profiles, with reductions in leptin and increases in adiponectin, which are known to influence cartilage metabolism and inflammatory pathways [30].

The metabolic effects of bariatric surgery may also influence the progression of structural joint damage. Some studies have reported increases in joint space width and improvements in subchondral bone microarchitecture after surgery, suggesting potential disease‐modifying properties [26, 27]. Rishi et al. (2018) observed that bariatric surgery delayed the need for total knee arthroplasty in patients with morbid obesity and symptomatic OA, supporting the hypothesis that weight loss may alter the natural history of disease progression [31]. Although these findings are encouraging, the long‐term structural effects remain insufficiently characterized due to limited longitudinal imaging data.

The present findings have important clinical implications. First, they support the consideration of bariatric surgery as part of a comprehensive management strategy for obese patients with symptomatic OA, particularly those with severe obesity refractory to lifestyle and pharmacologic interventions. In addition to improving metabolic health, bariatric surgery may reduce pain and enhance physical function, thereby improving overall quality of life and physical activity capacity. Second, bariatric surgery may serve as a bridge to definitive orthopedic interventions. Several studies have demonstrated that substantial weight loss prior to total knee or hip arthroplasty reduces perioperative risk and improves surgical candidacy. The integration of bariatric and orthopedic care within multidisciplinary frameworks may therefore optimize outcomes for patients with coexisting obesity and OA.

## 5 Limitations

This study has several limitations. Most included studies were observational, introducing potential confounding and selection bias. Variability in study design, sample size, and surgical techniques contributed to significant heterogeneity across pooled analyses. Outcome measures and follow‐up durations were not standardized, limiting direct comparability between studies. Additionally, radiographic or imaging‐based assessments of structural joint changes were infrequently reported, restricting conclusions about disease modification. Publication bias cannot be entirely excluded despite formal assessment. Finally, the limited number of long‐term studies reduces confidence in the durability of the observed improvements in osteoarthritis symptoms after bariatric surgery. A further important limitation is the absence of data linking the magnitude of weight loss to the degree of symptomatic improvement. None of the included studies presented study‐level or participant‐level estimates quantifying the association between weight change (e.g., absolute BMI reduction or %EWL) and change in OA diagnosis or WOMAC scores. Reporting such data will be critical to inform clinical decision‐making and to establish thresholds of weight loss that meaningfully alter OA symptoms or progression. An important consideration in interpreting these findings is the heterogeneity of bariatric surgical techniques included across studies. Although laparoscopic sleeve gastrectomy, Roux‐en‐Y gastric bypass, and laparoscopic adjustable gastric banding differ in their metabolic effects and typical weight loss trajectories, the available literature did not permit procedure‐specific comparative analyses.

## 6 Conclusions

Bariatric surgery is associated with significant improvements in osteoarthritis‐related pain, stiffness, and physical function, and with a reduced likelihood of a clinical osteoarthritis diagnosis after surgery. These findings primarily reflect symptomatic and functional improvement, as the available evidence is largely observational, heterogeneous, symptom‐based, and lacks standardized radiographic assessment or analysis of dose–response relationships between weight loss and osteoarthritis outcomes. Consequently, although bariatric surgery appears beneficial for symptom relief in patients with obesity and osteoarthritis, its effects on structural disease modification remain uncertain and warrant further prospective investigation.

## Author Contributions

**Mohammed Mana Mohammed Alqahtani:** conceptualization, investigation, methodology, writing – original draft, writing – review and editing. **António Raposo:** methodology, investigation, writing – review and editing, visualization, supervision. **Abdullah Mohammed Saeed Alshalaan:** investigation, writing – original draft, writing – review and editing, methodology. **Saeed Mohammed Saad Asiri:** investigation, writing – review and editing. **Jaber Salman Yazeed Alfaifi:** investigation, writing – review and editing. **Saleh Mohammed Saleh Alqahtani:** investigation, writing – review and editing. **Rawan Asad Salameh Shawabkeh:** investigation, writing – review and editing. **Afnan Ali Saeed Abonukhaa:** investigation, writing – review and editing. **Sara Ahmed Elmahadi Ahmed:** investigation, writing – review and editing. **Maha Hassan Ali Asiri:** investigation, writing – review and editing. **Mohammad Abdullah Saeed Almastoor:** investigation, writing – review and editing. **Ariana Saraiva:** investigation, writing – review and editing. **Ashwag Saleh Alsharidah:** investigation, writing – review and editing. **Ibrahim Yahya Al Yasin:** investigation, writing – review and editing. **Najim Z. Alshahrani:** conceptualization, investigation, methodology, writing – original draft, writing – review and editing, visualization, supervision.

## Funding

The authors have nothing to report.

## Consent

The authors have nothing to report.

## Conflicts of Interest

The authors declare no conflicts of interest.

## Supporting information


Supporting Information S1

## Data Availability

Data sharing is not applicable to this article as no datasets were generated or analyzed during this study.
