# Supplementary material for: Effect of Bariatric Surgery on Osteoarthritis‐Related Pain and Function: A Systematic Review and Meta‐Analysis
Source: World J Surg. 2026 Jan 21;50(3):513–22. doi: 10.1002/wjs.70236 (PMC13006778; doi:10.1002/wjs.70236)
Supplement: Supplementary file 1 — Supporting Information S1 [file WJS-50-513-s001.docx]

**Supplementary Materials**

PubMed

(("Bariatric Surgery"[Mesh] OR "Gastric Bypass"[Mesh] OR "Gastrectomy"[Mesh] OR "Metabolic Surgery" OR "Weight Loss Surgery" OR "Obesity Surgery" OR "Laparoscopic Sleeve Gastrectomy" OR "Sleeve Gastrectomy" OR "Roux-en-Y Gastric Bypass" OR "Laparoscopic Adjustable Gastric Banding" OR "Gastric Banding"))

AND

(("Osteoarthritis"[Mesh] OR "Knee Osteoarthritis" OR "Hip Osteoarthritis" OR "Degenerative Joint Disease" OR "Arthrosis" OR "Joint Pain"))

Scopus

(TITLE-ABS-KEY("bariatric surgery" OR "metabolic surgery" OR "weight loss surgery" OR "obesity surgery" OR "sleeve gastrectomy" OR "laparoscopic sleeve gastrectomy" OR "gastric bypass" OR "roux-en-y gastric bypass" OR "laparoscopic adjustable gastric banding" OR "gastric banding"))

AND

(TITLE-ABS-KEY("osteoarthritis" OR "knee osteoarthritis" OR "hip osteoarthritis" OR "degenerative joint disease" OR "arthrosis" OR "joint pain"))

WOS

TS=("bariatric surgery" OR "metabolic surgery" OR "weight loss surgery" OR "obesity surgery" OR "gastric bypass" OR "roux-en-y gastric bypass" OR "sleeve gastrectomy" OR "laparoscopic sleeve gastrectomy" OR "gastric banding" OR "laparoscopic adjustable gastric banding")

AND

TS=("osteoarthritis" OR "knee osteoarthritis" OR "hip osteoarthritis" OR "degenerative joint disease" OR "arthrosis" OR "joint pain")

Cochrane

([mh "Bariatric Surgery"] OR [mh "Gastric Bypass"] OR "bariatric surgery" OR "metabolic surgery" OR "weight loss surgery" OR "obesity surgery" OR "sleeve gastrectomy" OR "gastric bypass" OR "roux-en-y gastric bypass" OR "gastric banding" OR "laparoscopic adjustable gastric banding")

AND

([mh "Osteoarthritis"] OR "osteoarthritis" OR "knee osteoarthritis" OR "hip osteoarthritis" OR "degenerative joint disease" OR "arthrosis" OR "joint pain")
